# Early Events in Foamy Virus—Host Interaction and Intracellular Trafficking

**DOI:** 10.3390/v5041055

**Published:** 2013-04-08

**Authors:** Ursula Berka, Martin Volker Hamann, Dirk Lindemann

**Affiliations:** 1 Institute of Virology, Medical Faculty―Carl Gustav Carus, Technische Universität Dresden, Fetscherstr. 74, Dresden 01307, Germany; E-Mails: Ursula.Berka@tu-dresden.de (U.B.); Martin.Hamann@tu-dresden.de(M.V.H.); 2 DFG-Center for Regenerative Therapies Dresden (CRTD)—Cluster of Excellence, Biotechnology Center, Technische Universität Dresden, Fetscherstr. 105, Dresden 01307, Germany

**Keywords:** foamy virus, entry, trafficking

## Abstract

Here we review viral and cellular requirements for entry and intracellular trafficking of foamy viruses (FVs) resulting in integration of viral sequences into the host cell genome. The virus encoded glycoprotein harbors all essential viral determinants, which are involved in absorption to the host membrane and triggering the uptake of virus particles. However, only recently light was shed on some details of FV’s interaction with its host cell receptor(s). Latest studies indicate glycosaminoglycans of cellular proteoglycans, particularly heparan sulfate, to be of utmost importance. In a species-specific manner FVs encounter endogenous machineries of the target cell, which are in some cases exploited for fusion and further egress into the cytosol. Mostly triggered by pH-dependent endocytosis, viral and cellular membranes fuse and release naked FV capsids into the cytoplasm. Intact FV capsids are then shuttled along microtubules and are found to accumulate nearby the centrosome where they can remain in a latent state for extended time periods. Depending on the host cell cycle status, FV capsids finally disassemble and, by still poorly characterized mechanisms, the preintegration complex gets access to the host cell chromatin. Host cell mitosis finally allows for viral genome integration, ultimately starting a new round of viral replication.

## 1. Introduction

**Figure 1:**
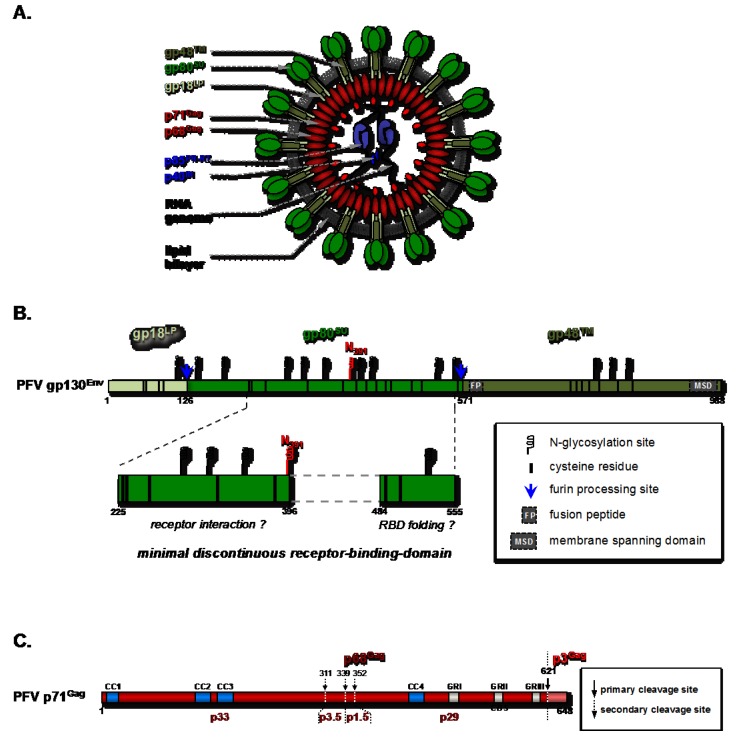
Particle composition and structural protein organization of PFV. (**A**) Schematic outline of a PFV particle. Two copies of the single-stranded RNA genome are encapsidated, which in the virus-producing cell is already reverse transcribed into DNA in a significant fraction of virions. The viral nucleic acids as well as pol-encoded protease-reverse-transcriptase(PR-RT) and integrase enzymes are surrounded by a protein shell, composed of gag-encoded capsid protein precursor (p71^Gag^) and its large processing product (p68^Gag^). During budding a host-cell derived lipid bilayer is acquired, which harbors the mature viral glycoprotein complex containing leader peptide (gp18^LP^), surface (gp80^SU^), and transmembrane (gp48^TM^) subunits. (**B**) Schematic organization of the PFV glycoprotein precursor protein. The individual mature glycoprotein subunits are indicated and color-coded. Below the minimal, discontinuous receptor-binding-domain characterized within the SU subunit is enlarged. N-glycosylation sites, cysteine residues and furin processing sites are indicated. Numbers given below indicate amino acid positions. (**C**) Schematic organization of the PFV capsid protein precursor protein. The individual mature products derived by viral protease-mediated cleavage at primary or secondary processing sites (numbers given above indicate amino acid positions) are indicated. Functional domains characterized within the Gag protein are marked. CC: putative coiled-coil motif; GR: glycine-arginine rich box; CBS: chromatin binding site.

Successful attachment to and entry into host cells are essential events for initiation of viral replication. Foamy viruses (FV) are characterized by an exceptionally broad tissue tropism that differs from any other retrovirus [1,2,3]. The spumaretroviral envelope protein (Env) harbors essential features that allow for absorption, uptake and fusogenic release of capsids into the cytoplasm. In comparison to other retroviral envelope proteins FV glycoproteins undergo a highly unusual biosynthesis as the precursor (gp130^Env^ for prototype FV, PFV) is only posttranslationally cleaved by cellular furin-like proteases (reviewed in [10]). Env precursor processing then culminates into an N-terminal signal or leader peptide (LP, gp18^LP^ for PFV), a central surface (SU, gp80^SU^ for PFV) and a C-terminal transmembrane subunit (TM, gp48^TM^ for PFV) [4]. Characteristically for FVs, three copies of each of the three mature subunits of the viral glycoprotein assemble in heterotrimeric complexes incorporated into the membrane of released virions (Figure 1A) [5]. The FV Env trimers thereby appear to cluster into metastable hexamers with a central depression, thus giving the characteristic prominent extracellular spike structures observed on electron micrographs of FV particles [5]. Currently it is unknown whether this hexameric organization of the FV Env glycoprotein complexes is essential for host cell receptor recognition, as cellular attachment and entry factors are poorly characterized and non-permissive cells were only recently identified [6,7,8]. The first part of this review will focus on our current knowledge of molecular determinants of initial virus-host interactions that allow for FV attachment and glycoprotein-dependent release of capsids into the cytoplasm. 

The second part summarizes information available on subsequent steps of viral replication that ultimately lead to insertion of the viral DNA into the host cell genome. Upon crossing the first physical barrier imposed by the cell membrane and escape of capsids into the cytosol FVs face some daunting challenges. Unique among retroviruses is entry of different kinds of FV particles, containing either a DNA or RNA genome, as a consequence of the reverse transcription initiation in a significant fraction of virions already in the virus producing cell (reviewed in [9,10]). To eventually deliver the viral genome to its final destination—the nucleus—the capsids first migrate through the cytoplasm of the host cell using cytoskeletal structures and motor protein complexes [11,12,13,14]. Upon accumulation at the microtubule organizing center (MTOC), FV capsids require poorly characterized cellular cues for uncoating and formation of the preintegration complex (PIC) [11,15,16]. Cell-cycle dependent integration of FV genomes is only observed upon mitosis and nuclear membrane breakdown [11,17,18,19].

## 2. Early Events in Virus-Host Interaction: Attachment and Entry

### 2.1. Molecular Determinants of the Foamy Viral Glycoprotein for Attachment and Entry

#### 2.1.1. FV-Env Mediated Superinfection Resistance

In the 1970s, early investigations on retroviral glycoproteins revealed inhibition of secondary exogenous infections by binding of membrane-anchored or intracellular expressed glycoproteins to cellular receptors, a phenomenon known as superinfection exclusion or superinfection resistance (SIR) (reviewed in [20,21]). Whether SIR of target cells particularly involves the down-regulation of receptor expression or rather the masking of binding pockets is still debatable and likely species-specific. However, it provides means to examine host receptor usage of different virus species without knowledge of molecular details on the specific receptors involved.

Moebes *et al.* first reported indications of SIR also for FVs as PFV-dependent marker gene transfer was inhibited in PFV Env wt expressing baby hamster kidney (BHK) cells [22]. Subsequently, SIR of PFV Env expressing cells towards other FV species or retroviral vectors pseudotyped with glycoproteins of primate and non-primate FV species, but not murine leukemia virus (MLV) Env or vesicular stomatitis virus glycoprotein pseudotypes was demonstrated [1,23]. Thus, these data clearly indicate that different FV species, independently of genetic clustering into primate and non-primate FVs, use common molecule(s) for attachment and/or entry, at least into the cells (hamster, BHK and human HT1080) examined. 

The early indications of FV Env-dependent SIR were further strengthened by additional findings that glycoprotein subunit processing, efficient cell surface transport and membrane anchoring are of utmost importance for PFV Env-mediated SIR [23]. Strikingly, whereas secretion of the MLV Env SU domain or its receptor-binding-domain (RBD) readily induces SIR, neither secreted monomeric PFV SU nor alternatively membrane anchored PFV SU domain was sufficient for SIR induction [23]. PFV Env-mediated SIR rather depended on the extracellular domains of both the SU and the TM subunits and only PFV glycoproteins capable of correct gp130^Env^ precursor processing blocked challenge infections. Therefore, oligomeric organization and structure of the PFV Env glycoprotein complex appear to be important for interactions with the host cell molecule(s) essential for mediating SIR. Additionally, FV glycoprotein mutants with decreased cell surface transport and/or membrane expression were incapable of inducing resistance to challenge infection [23]. It is yet unclear whether such mutants undergo misleading intracellular trafficking and thus lack posttranslational modifications interfering with its proper folding and/or whether their inefficient targeting to the trans Golgi network might disable efficient interaction with target receptors destined for cell surface expression that is required for SIR. 

#### 2.1.2. The FV Receptor-Binding-Domain and Its Functional Dependence on Post-Translational Modifications

The SIR induction upon expression of the FV glycoprotein alone, and the identical tropism of FVs to retroviruses pseudotyped with FV Env glycoproteins, suggest that, similar to other retroviruses, the main determinants of FV host range and specific entry into target cells are located in the viral Env protein. To understand which particular viral motif(s) and/or structures of the FV glycoprotein are essentially involved in receptor binding, various portions of the extracellular domain of PFV or chimpanzee FV (SFVcpz) Env were linked to IgG heavy chain Fc regions and recombinant immunoadhesins were analyzed for their specific target cell-binding capacity by flow cytometry [24,25]. These studies revealed that the LP and TM domains are dispensable for host cell binding and the putative receptor-binding-domain (RBD) is located in the Env SU subunit. This is in general agreement with receptor binding of retroviruses via their Env SU domain (reviewed in [26]). Upon N-, C-, but also internal deletion analysis of the PFV SU domain, a minimal, discontinuous RBD region spanning amino acids 225 to 396 and 484 to 555 was defined (Figure 1B) [25]. Similar to human immunodeficiency virus 1 (HIV-1), the PFV RBD is located in the C-terminal part of the SU subunit, whereas MLV harbors an N-terminally encoded RBD [27,28,29,30,31]. 

Notably, immunoadhesins containing either the SFVcpz or PFV Env SU bound dose-dependently to FV permissive cells [24,25]. For SFVcpz immunoadhesins specific host cell recognition was abolished upon incubation with neutralizing serum or detergents [24]. In contrast, such binding of SFVcpz immunoadhesins to host cell did not interfere with FV infection. This indicates inefficient neutralization of sensitive if not multivalent cellular epitopes by rather weakly binding monomeric SU-domains. Thus, in contrast to other retroviruses, FV-mediated SIR might require a rather high affinity, quantity and/or multivalent interaction of cellular receptors with viral components [24].

The number and position of N-glycosylation sites in FV Env differ from HIV-1 and MLV [32,33]. However, post-translational modifications such as the attachment of oligosaccharides to viral Env asparagine residues are not only known to promote proper folding or intracellular trafficking but also to ensure for specific interaction with receptor molecules (reviewed in [34]). Indeed, high-affinity binding of PFV Env to target cells is compromised if the natural N-glycosylation site 8 (N_391_, Figure 1B) is mutated or even when it is shifted only a few amino acids towards the C-terminus of the FV SU domain [25]. This indicates an essential if not direct involvement of N-glycosylation site 8 and surrounding sequences in PFV receptor interaction. With this glycosylation site being highly conserved among 15 different FV species, this might be a matter of principle in FV virus receptor interaction [25,32].

Beside glycosylation, intra-chain disulfide bonds might segregate functional motifs within viral glycoproteins (reviewed in [26]). Three-dimensional modeling of disulfide bond arrangements within the Hepatitis C virus (HCV) glycoprotein helped to predict the folding of its RBD and conformational changes induced upon viral fusion [35]. More recently, entry and fusion competence of the HCV glycoprotein heterodimer E1E2 was shown to involve conserved cysteine residues in the E2 but not mandatorily in the E1 glycoprotein [36,37]. Similar to other retrovirus genera, the cysteine residues of glycoproteins from different FV species are evolutionarily highly conserved (Figure 1B) [25,38]. 

However, unlike HCV or other retroviruses, the particular pattern of intra-chain disulfide bonds has not yet been characterized in detail for any FV glycoprotein. Mutational analysis of the PFV Env indicated that all cysteine residues in the N-terminal part of the SU domain are essential for RBD formation whereas only few of the C-terminal part are absolutely required [25]. Although, mutation of individual C-terminal cysteine residues reduced binding of PFV SU-immunoadhesins, residual binding activity was retained. Taken together these data are in line with the characterization of the RBD by deletion analysis and suggest the N-terminal part of the bipartite RBD is important for receptor binding whereas the C-terminal part appears to stabilize or enhances RBD folding. 

### 2.2. Cellular Determinants for FV Attachment and Uptake

Independently of characterization of essential viral determinants, the nature of the particular host molecule(s) involved in FV attachment and/or entry is still understood very poorly. The broad host range of FVs and the lack of non-permissive cell lines severely limited studies on host cell requirements until recently. Herchenröder *et al.* already proposed the use of rather non-perfectly conserved cellular FV receptor(s) and a more tolerant mechanism of viral binding and uptake as an explanation of the unusual broad host range of FVs [24]. For example, SFVcpz EnvSU-Ig chimera bound to host surface molecule(s) in a detergent-sensitive but trypsin-insensitive fashion, which was proposed to point to glycosidic residues, a component of numerous membrane proteins or lipids, to be a potential receptor. Furthermore, the biphasic binding curve of SFVcpz immunoadhesin to host cells might be either a result of a weak affinity of the immunoadhesin and/ or the involvement of at least one additional cellular factor that allows for particularly high-affinity attachment [24]. 

Flow cytometry based virus binding assays are another way to examine the attachment of viruses to host cells and interaction with cellular receptors. They allow quantification of retrovirus binding and, in respect to the transduction efficiency, assessing the expression of functional receptor molecules [39]. Only recently, Stirnnagel *et al.* applied such an approach to study the interaction of infectious FV vector particles, containing capsids tagged with fluorescent proteins, with host cells [8]. In this study two cell lines (the zebrafish cell line Pac2 and the human erythorid precursor cell line G1E-ER4) were identified for the first time that appear to be non-permissive to retroviral vector-mediated marker gene transfer via the PFV glycoprotein. The non-permissive phenotype was independent of the particular capsid used as both FV and HIV-1 particles pseudotyped with PFV Env were incapable to productively transduce these cell lines. Strikingly, these cell lines still displayed PFV Env dependent attachment of fluorescently labeled virions but virus uptake and vector genome expression were blocked. Thus, in line with the original notion of Herchenröder *et al.* [24], FVs might use different attachment and entry receptor molecules.

**Figure 2:**
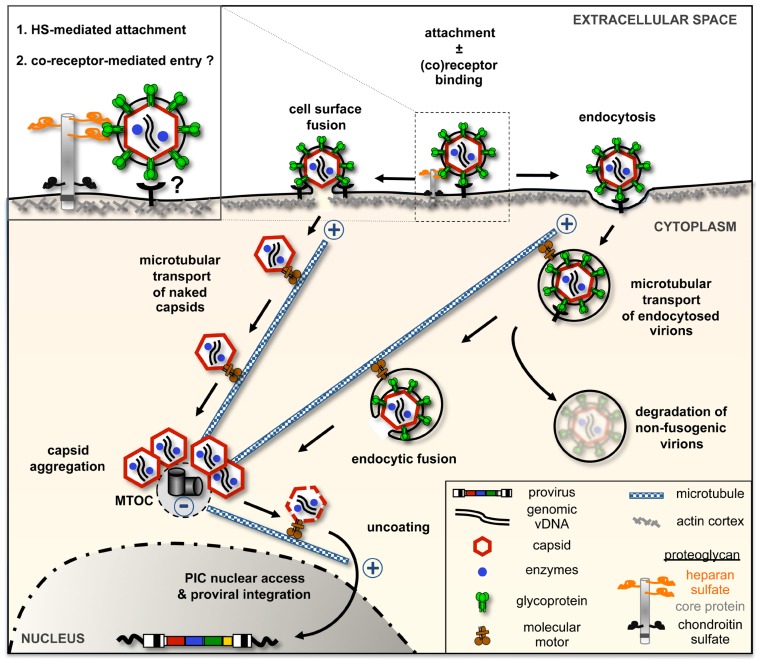
Model of foamy virus (FV) glycoprotein-dependent entry into host cells. FV particle attachment to the surface of host cells is strongly enhanced by heparan sulfate (HS) chains of proteoglycans. Whether virion uptake and fusion of viral and cellular lipid membranes require yet unidentified additional entry receptor(s) remains unclear. Capsids of most FV species appear to get cytoplasmic access by endocytosis and glycoprotein-mediated, pH-dependent fusion process at internal membranes. PFV entry is unique as a significant portion of capsids is released into the cytoplasm by fusion of virions with the plasma membrane. Cytoplasmic capsids are actively transported towards the nucleus along microtubules on dynein motor protein complexes. Naked FV capsids accumulate at the MTOC were they remain in a latent state for extended periods of time until the host cell transits into mitosis. Further capsid uncoating appears to proceed in a cell-cycle-dependent manner, requiring cellular proteases and potential enhancement by viral protease activity. FV preintegration complex (PIC) access to the cellular chromatin requires nuclear membrane breakdown during mitosis. During mitosis FV PICs are tethered to cellular chromosomes via the Gag localized chromatin binding site (CBS).

Extracellular matrix (ECM) components are known to assist the infection of a variety of virus including herpes simplex virus, HIV-1, adenovirus and hepatitis C virus (reviewed in [40]). Several recent reports have addressed the involvement of components of the ECM or cellular membranes in FV binding and uptake (Figure 2) [6,7,8]. In the scope of the experiments performed, Stirnnagel *et al.* found no indication for cellular lipids contributing to PFV attachment. Unlike VSV-G containing particles, no binding of retroviral particles containing PFV Env to lipids extracted from Hela cells or various synthetic lipids was detectable [8]. In contrast, both binding of as well as transduction by PFV Env containing vector particles were shown to be enhanced by proteoglycans as demonstrated using parental mouse L-cells and a proteoglycan-deficient subclone thereof (SOG9). 

The contribution of proteoglycans, and different glycosaminoglycans (GAG) in particular, was examined into greater detail in two additional studies [6,7]. The observation that overnight incubation of target cells on fibronectin, which was associated with a downregulation of heparan sulfate (HS) from the cell surface, strongly impaired PFV vector transduction, led Nasimuzzaman and Persons to investigate the role of this ECM component for FV infection [6]. They observed a correlation of HS surface expression with viral susceptibility, a low permissivity of cells deficient in HS synthesis or cells having HS enzymatically removed prior to transduction, and a strong enhancement of transduction efficiency by ectopic expression of heparan sulfated syndecan-1 in cells naturally not expressing HS. 

Similarly, Plochmann and colleagues reported a correlation of HS but not syndecan-1 cell surface expression on different cell lines with viral susceptibility, the diminishment of PFV infection by enzymatic removal of HS on target cells, as well as the severe impairment of PFV and FFV transduction by preincubation with Heparin but not other ECM components [7]. Interestingly, they demonstrated a utilization of the FV Env-HS interaction for affinity purification of PFV vector particles by Heparin columns. 

Thus, there are clear indications that cell surface HS serves as an key attachment factor for FVs and might represent the scaffold initiating multimeric interactions, as proposed by Herchenröder *et al.*, that result in clustering and activation of signaling pathways and eventually viral uptake [24]. A total lack of HS, however, does not completely abolish FV infection. It remains to be seen whether FV can use other attachment factors, although other GAG tested so far, such as chondroitin sulfate A, B, and C; hyaluronic acid; keratan sulfate; and N-acetylneuraminic acid, did not diminish PFV transduction [7]. Furthermore, these studies suggest that FV might require yet unidentified cellular entry receptors for viral fusion and release of capsids into the cytoplasm. However, they do not exclude an alternative mechanism combining the triggering of conformational changes by binding to attachment factors such as HS and a subsequent pH change to be sufficient for PFV entry.

### 2.3. FV Env-Dependent Fusogenic Release of Viral Capsids

Whereas the retroviral Env SU domain accounts for receptor binding, the TM domain is generally agreed to trigger membrane fusion (reviewed in [26]). A hydrophobic sequence downstream of the discontinuous PFV RBD (fusion peptide, FP; Figure 1B) likely contributes to merging of viral and cellular membranes, but shares no sequence similarity with known retroviral N-terminal fusion peptides [32,41,42]. An intact PFV TM membrane spanning domain (MSD) and the presence of Gag protein enhance syncytia formation by fusion from without [43]. However, during entry, viral envelope and capsid segregate and naked capsids are released into the cytoplasm of infected cells (Figure 2). 

Processing of the FV Env precursor between SU and TM, but not LP and SU subunits, is required for viral infectivity (Figure 1B) [43,44,45,46]. Particularly, amino acid exchanges R_571_T in PFV and RKRR_570-573_AAEA in SFV_mac_ Env, respectively, resulted in inactivation of the Env SU-TM subunit furin cleavage site and rendered virions fusion-incompetent and severely reduce infection [14,43,44]. Monitoring entry of PFV virions, harboring both eGFP-tagged capsid and mCherry-tagged Env proteins by live-cell imaging allowed the analysis of binding, uptake and penetration processes in individual host cells [14]. Release of the majority of capsids into the cytoplasm by FV glycoprotein-mediated fusion within the first six hours after initiation of uptake was suggested thereby confirming previous studies using fixed samples of FV infected host cells [11,13]. Virions that failed to succeed in productive membrane fusion within this time period, or were fusion-incompetent due to inactivation of the SU/TM processing site, appeared to be rapidly cleared by degradation, probably within lysosomes (Figure 2) [14]. 

Notably, differences in the kinetics of the appearance of capsids in the cytoplasm were observed, in dependence of the type of FV glycoprotein used [14]. Quantitative monitoring of the fraction of individual viruses containing both Env and capsid signals as a function of time demonstrated that PFV Env containing virions fused within the first few minutes, whereas fusion of SFVmac Env containing virions was less frequent and observed over the entire 90 min measured. Consistent with different fusion activities of PFV and SFVmac glycoproteins, syncytia formation by a fusion from without mechanism was observed for cells coated with PFV Env but not SFVmac Env containing particles [14,47]. PFV is thus proposed to possess a fusion-active state already if bound to the plasma membrane whereas SFVmac fusion might be restricted to intracellular organelles (Figure 2). 

In line with this, an earlier study demonstrated a pH-stimulated fusion process mediated by glycoproteins of different FV species [47]. Strikingly, PFV Env was the only glycoprotein tested that displayed a relatively high fusion activity at neutral pH whereas the glycoproteins of simian, equine, feline or bovine origin, respectively, possess fusion-competence predominantly around pH 5.5. Taken together this might explain the different fusion kinetic of PFV compared to SFV as the latter rather depends on transport to and maturation of endosomal compartments. 

Furthermore, PFV Env was less sensitive to lysomotrophic agents that modify endocytic pH and thus block infection via endocytic uptake routes [14,47]. Independent of the target cell, all FV species are characterized by a failure of chloroquine, a weak base decreasing endo- and lysosomal acidification, to inhibit viral entry and/ or fusion. Whereas this feature substantially differs from the requirements described for VSV uptake, all FVs—except PFV—might therefore use a common pH-dependent, endocytic entry pathway (Figure 2). 

## 3. Post Fusion Events in FV Infection: Intracellular Trafficking, Disassembly and Formation of the Preintregration Complex

### 3.1. FV Capsid Trafficking towards the Microtubule-Organizing Center

As described above, fusion of viral and cellular membranes, at the cell surface or after endosomal uptake, ultimately leads to the release of naked FV capsids into the cytoplasm (Figure 2) [11,13,14]. The virions that escape degradation face now the daunting task of transporting their genomic information towards the nucleus before eventually integrating their genome into the host DNA. Imagining the cytosol as a densely packaged lumen containing a vast variety of cellular proteins, organelles and metabolites, pure diffusion of the capsid towards its destination seems unlikely. Instead, hijacking the cytoskeleton for capsid transport, in particular the microtubule (MT) network, is a phenomenon observed for many viruses (reviewed in [48]). 

Early studies on FV entry reported a pericentriolar accumulation of naked FV capsids, that was sensitive to nocodazole treatment, an agent that prevents microtubule polymerization, thus highlighting microtubules as a prime route for trafficking of FV capsids [12,13]. This view was supported by recent live cell imaging analysis of fluorescently labeled FV capsids in individual infected cells that, once released into the cytoplasm upon fusion, migrate towards the center of the cell [14]. The endpoint of FV capsid translocation is the MTOC (Figure 2) [12,13,14]. Electron micrographs (EM) revealed the presence of naked capsids in direct proximity of the MTOC [12]. However, whereas Gag proteins can be detected in the nucleus, entire capsids were never observed within the nucleus or close to nuclear pores, indicating that a disassembly step has to commence before nuclear entry of the viral genome [12,13,49,50]. FV capsid trafficking to the centrosome was fast, as already 5 h post infection almost all capsids were accumulated at this tubulin-nucleating center [14,16].

Retrograde movement along microtubules, meaning translocation towards the MTOC, is mediated by dynein motor proteins, which form a complex with the dynactin cofactor (dynein/dynactin-complex) [51,52]. This complex is involved in numerous cellular functions such as cell division and intracellular transport. Interestingly, PFV capsid transport was abolished by over-expression of the central coiled-coiled domain of p150^Glued^, a dynactin sidearm subunit [12]. This indicates the requirement of dynein motor complexes for FV Gag transport. Consistent with this finding, the dynein light chain 8 (LC8) was coprecipitated with a C-terminally truncated PFV Gag protein and vice versa [12]. 

Interaction of PFV Gag with LC8 involves one out of four putative coiled-coil domains (CC3, aa 160–180) (Figure 1C) since Gag mutants with abolished coiled-structure showed no centrosomal localization upon *de novo* expression in host cells [12]. Furthermore, proviral constructs with mutations in this Gag CC domain displayed normal expression and particle release characteristics, while viral infectivity was strongly diminished, although not completely abolished [12]. Since these mutant virions showed residual infectivity FVs might utilize additional or alternative intracellular transport routes. Early work from Giron and colleagues suggested an interaction of Gag with cellular actin, which would allow access to yet another elaborated cytoskeleton network [53]. Indeed, HIV-1 extensively hijacks the actin skeleton for various steps in its replication cycle (reviewed in [54]), making it likely for FVs to utilize it in one or the other way as well. 

### 3.2. Cell Cycle Dependence and Essential Components for Genome Integration

In contrast to lentiviruses but in common with gammaretroviruses, FVs are not able to efficiently infect non-proliferating cells [11,17,18,19]. Neither G1/S nor G2-phase arrested cells were efficiently infected by FVs or transduced with FV vectors, indicating that mitosis is essential for integration of viral genomes into the host cell chromosomes [17,18,19]. Similarly, G0-phase arrested primary fibroblasts or peripheral T cells challenged with FV could not be productively infected [11]. In these cells intact viral capsids accumulated at the MTOC after their release into the cytoplasm, but remained there in a latent state (Figure 2). Even several weeks later these capsids were capable to resume viral replication cycle leading to a productive infection once the host cell undergoes mitosis upon reentry into the cell cycle. 

With the onset of mitosis capsid uncoating and formation of the preintegration complex (PIC) appear to commence. The molecular cues signaling the virus that mitosis is about to start are currently unknown. Both, the MTOC and the associated centrosome are intimately connected to cell cycle regulation and hence display a junction for numerous signal pathways. It is likely that proteins orchestrating the cell cycle also recognize viral components and mediate essential reactions that promote FV capsid uncoating which might be a rate-limiting step in viral infection. Whether capsid disassembly is a single or multi-step process, and which particular cellular cues and viral components are essential or contribute to this process, is largely unknown (see below) [11,15]. 

The components of the FV PIC are not well characterized to date. Predictably, the integrase (IN) and the viral DNA genome are essential components of the PIC. The former one was suggested to play an active role in SFVmac Gag and viral DNA (vDNA) transport into the nucleus as it contains a nuclear localization signal (NLS) [18,55,56,57]. Interestingly, SFVmac vDNA was found in the nucleus of G1/S arrested cells in a non-integrated episomal state, but no viral expression was detectable [18]. Upon entry into mitosis viral replication proceeded and vDNA integration and gene expression was detectable. Thus FV genomes appear to translocate to the nucleus in interphase cells, at least in case of SFVmac, but remain in latent, non-integrated state preventing viral gene expression.

A 13 residue motif, termed chromatin binding site (CBS), could be mapped to the N-terminal region of GR-box II, one of three glycine/arginine (GR)-rich boxes located in the C-terminus of PFV Gag (Figure 1C). The CBS is responsible for tethering of FV Gag to host cell chromatin [58]. Directly adjacent to the CBS in Gag a sequence with putative NLS activity was described originally as being responsible for the transient nuclear localization of the Gag protein in FV infected cells [50,58,59]. 

However, recent analysis of different steps of FV replication by live cell imaging and fluorescent protein-tagged structural proteins demonstrated that FV Gag is indeed not actively transported into the nucleus of interphase cells [14,60]. FV Gag only gets access to the host chromatin upon nuclear membrane breakdown during mitosis. An additional GR-rich motif, GR-box I, was previously shown to be important for viral nucleic acid binding, although the contribution of individual GR-boxes to RNA packaging is discussed controversial (Figure 1C) [59,61,62]. 

The synergy of chromatin tethering and nucleic acid binding features of FV Gag suggest a role of this viral protein as a bridging molecule between viral DNA and host chromosomes, which probably promotes the integration reaction [58]. Fluorescence *in-situ* hybridization (FISH) experiments with virus specific probes confirmed the co-localization of Gag and viral DNA [58]. Furthermore, co-immunoprecipitation studies revealed an interaction of PFV Gag with histones H2A/H2B, identifying at least one protein complex that interacts with the viral components. However, whether other cellular proteins contribute to integration and selection of the integration site remains unclear. 

### 3.3. Capsid Disassembly—A Concerted Process Involving Cellular and Viral Enzymes?

Many questions remain open on the mechanism of uncoating of FVs as of other retroviruses such as HIV (reviewed in [63,64]). In case of HIV-1, capsids appear to be quite instable and uncoating commences within one hour after their release into the cytosol. In contrast, MLV capsid seems to be more stable and uncoating is thought to take place only after nuclear entry following mitosis. Recent data on HIV-1 suggest that uncoating and reverse transcription proceed in parallel and may influence each other. The subcellular location of HIV-1 uncoating within host cells is presently unclear. Early work indicated that it commences immediately following fusogenic release of capsids and supported an uncoating in the cytoplasm. More recent evidence implies the nuclear pore complexes to be the place of both disassembly and release of the preintegration complex. 

Foamy viruses differ in several aspects compared to their orthoretroviral counterparts. In the sense of genome reverse transcription, FVs undergo this reaction to a large extent late in the replication cycle, still in the virus producing cell [22,65]. Hence FV capsids already contain large amounts of viral DNA genome as they enter the target cell and approach the nucleus. Additional genome reverse transcription upon target cell entry of FVs is also observable and is thought to be important at low multiplicities of infection [66,67]. The viral capsid shields the viral nucleic acids as they are transported along the microtubules to the MTOC (Figure 2). 

Unlike other retroviruses, FV Gag proteins undergo only a very limited precursor processing by the *pol*-encoded viral protease (PR) during assembly and release (reviewed in [9,10]). Therefore, mature FV capsids entering host cells lack the canonical orthoretroviral-like matrix (MA), capsid (CA), and nucleocapsid (NC) subunit organization and are composed mainly of the Gag precursor (p71^Gag^ for PFV) and its large cleavage product (p68^Gag^ for PFV) (Figure 1A,C). Three additional (secondary) cleavage sites of the viral protease clustered in the center of the Gag precursor (at position 311, 339 and 352 in PFV p71^Gag^) have been described (Figure 1C) [68]. Interestingly, mutation of the first cleavage site at position 311 prevented the processing at the two downstream sites, which indicates a timely orchestrated process [68]. 

Pfrepper *et al.* proposed a utilization of these secondary Gag processing sites during disassembly of the capsid upon host cell entry as their inactivation in the proviral context resulted in non-infectious viruses, however, particle release of the mutants appeared to be reduced as well [68]. In a follow-up study of this initial observation Lehmann-Che and co-workers addressed the dependency of FV entry on viral PR activity [16]. They observed a similar phenotype for PR-deficient PFV particles being reconstituted with the large p68^Gag^ subunit *in trans* and a PFV Gag mutant with inactivated secondary Gag processing site at position 311 (I310E mutation). Both types of PFV mutants showed a similar particle release, initial uptake into host cells and accumulation of naked capsids at the MTOC as wild type PFV. However, particles of both PFV Gag mutants were non-infectious, and, unlike wild type PFV, subsequent nuclear localization of the Gag protein and viral genome was abolished. Most importantly, the appearance of a Gag cleavage product, derived by processing at the secondary cleavage site and observed over time in target cells infected with wild type PFV particles, but not other cleavage products, probably derived by processing of unknown cellular PRs, was absent in samples infected with both types of mutant PFV particles. This led to the conclusion that following accumulation of naked FV capsids at the MTOC during target cell entry, FV uncoating, release of the PIC and its nuclear localization require an essential proteolytic processing by the viral PR at Gag secondary cleavage sites. 

Though, this dogma of a viral PR-dependent FV uncoating was recently challenged by Hütter *et al.* [15]. It was demonstrated that infectious PFV particles with enzymatically inactive viral protease, derived from proviral and different replication-deficient vector system constructs, could be obtained if the capsids of the virions, unlike wild type, were composed of the large Gag subunit (p68^Gag^) only. This renders the viral PR activity as being not absolutely essential for FV uncoating. However, the infectivity of these PR-deficient virions was strongly impaired (app. 100-fold reduced compared to wild type). This might be a consequence of the reduced intra-particle reverse transcription observed for PFV particles composed of p68^Gag^ only, in combination with a reduced integration potential as no mature IN subunit is present due to failure of Pol precursor processing. However, the study by Hütter *et al.* not formally excludes the possibility of further processing of the capsid by viral PR at the secondary Gag cleavage sites that might enhance viral uncoating [15,16].

## 4. Innate Sensing and Cellular Restriction Factors of FVs

Viruses not only exploit cellular processes and machineries for their replication. They also have to avoid or counteract antiviral responses, such as recognition by the innate immune system or cellular antiviral restriction factors, developed by the host during evolution. A striking feature of FVs is the discrepancy of strong cytopathic effects observed for replication *in vitro*, ultimately leading to the death of most infected cell types, and apparent apathogenicity of FV infections in natural hosts or zoonotically infected humans (reviewed in [69]). Whether this is the result of a coevolution of virus and host and reflects a rather balanced virus-induced immune response, potentially associated with a limitation of replication to superficial epithelial cells of the oral mucosa, remains to be seen [70].

Only in recent years, we are beginning to better understand how retroviruses are recognized and/or avoid detection by the innate immune system and which antiviral factors they encounter during target cell entry (reviewed in [71,72]). Innate immune system sensing of FV infections is poorly characterized. A failure of FVs to induce type I interferon (IFN-I) was suggested by early studies using primate or human cell lines [73,74,75]. A very recent study by Rua and colleagues, however, demonstrates that FVs are efficiently sensed by primary human hematopoietic cells [76]. Plasmacytoid dendritic cells (pDC), the main IFN-producing cell in the organism, appeared to be the major source of IFN-I production in human peripheral blood mononuclear cells (PBMC) stimulated with FV particles or FV infected cells. IFN-I induction did not require an enzymatically active FV reverse transcriptase indicating the viral RNA and not DNA also present in FV particles is the main trigger for innate immune system activation. In line with this, inhibitors of endosomal acidification, gene silencing and endosomal Toll-like receptor (TLR) antagonists strongly suggest FVs to be predominantly sensed by cellular TLR7 molecules recognizing viral genomes. As proposed by the authors, this activation of the innate immune responses may be involved in the control of viral replication in infected humans or natural hosts. 

In line with this finding previous studies reported a sensitivity of FV infection to IFN-I [74,75,77,78,79]. Indeed, several IFN-induced cellular gene products are known to interfere with FV replication in culture systems [77,78,80,81,82,83,84,85,86]. Though only tripartite motif protein 5α (TRIM5α) is implicated at restricting FVs during viral entry [85,86]. TRIM5α proteins, composed of RING, B-Box, coiled-coiled (RBCC) and B30.2 (or PRYSPRY) domains, inhibit replication of particular retroviruses in a species-specific manner (reviewed in [87,88]). The PRYSPRY domain appears to determine the main specificity by mediating the interaction of TRIM5α with the capsid domain (CA) domain of processed multimerized Gag proteins, forming a hexagonal lattice on top of the retroviral capsid. Orthoretroviral replication is arrested prior to reverse transcription by a dual function of TRIM5α. TRIM5α engagement of the retroviral capsids results in an aberrant uncoating of the capsid and concomitantly triggers a pattern recognition function that involves its E3 ubiquitin ligase activity, thus initiating an innate immune response within the infected host cell. 

Although FVs unlike orthoretroviruses have rather an immature capsid morphology, as a consequence of the limited Gag precursor processing (see above), various FV species examined showed a restriction by different TRIM5α proteins in a species-specific manner [85,86]. TRIM5α of New World monkeys (NWM) restricted PFV, FVs from Old World monkey (OWM) and some NWM FVs whereas replication of the non-primate feline FV (FFV) was inhibited by some TRIM5α proteins of Apes and NWMs [85,86]. On the cellular side the determinants of restriction specificity were located in the V domains of the PRYSPRY domain whereas the restricting viral determinant was mapped to the N-terminal part of the respective FV capsid proteins. [86].

Taken together, restriction of FVs by different TRIM5α proteins is consistent with the proposed ancient cospeciation of simian FVs and their hosts [89]. The TRIM5α restriction pattern within different NWM species suggest that during successful adaption to a new host species, each SFV has apparently evolved to minimize the detrimental impact of the particular TRIM5α protein encountered [85].

## 5. Conclusions

Significant progress in the understanding of FV entry processes has been achieved in the last two decades, revealing analogies to other retroviruses but also characterizing distinct features unique for FVs. A more detailed characterization of first cellular molecules important for attachment and/or uptake of FVs that may explain the broad host range conferred by FV glycoproteins is of particular interest. A main question still to be answered is whether single molecules such as HS and particular proteoglycan core proteins are sufficient to allow for FV binding, cell signaling activation and triggering FV Env's fusogenic activity or whether yet unknown additional factors are required. Identification of apparently non-permissive host cell lines and the possibility of analyzing FV entry on the single-cell level in real time might be helpful for these studies.

Likewise, our picture on the intracellular steps of FV entry, concerning capsid trafficking, uncoating and the formation of the PIC, is also far from complete. Further efforts are needed to foster our understanding on the transport pathways FV use to target the MTOC and how capsids engage particular motor proteins. FVs’ cell-cycle dependence and the need for host cell mitosis might point towards specific mitotic protein complexes essential for ongoing viral infection. However, the distinct proteins and their functions remain to be identified. 

Moreover, initial insights of FVs interaction with sensors and effectors of the innate immune system that have been unraveled recently might be of importance for developing concepts explaining the apparent apathogenicity of these viruses in their natural hosts and infected humans. 

In summary, expanding our knowledge on FV entry processes will aid our understanding on how this special type of retroviruses interacts with the host cell and will advance the development of FVs as vehicles for gene therapy purposes.
